# Human Embryonic Stem Cell Derived Mesenchymal Progenitors Express Cardiac Markers but Do Not Form Contractile Cardiomyocytes

**DOI:** 10.1371/journal.pone.0054524

**Published:** 2013-01-16

**Authors:** Christophe M. Raynaud, Najeeb Halabi, David A. Elliott, Jennifer Pasquier, Andrew G. Elefanty, Edouard G. Stanley, Arash Rafii

**Affiliations:** 1 Stem Cell and Microenvironment Laboratory, Weill Cornell Medical College in Qatar Education City, Qatar Foundation, Doha, Qatar; 2 Department of Genetic Medicine, Weill Cornell Medical College, New York, New York, United States of America; 3 Monash Immunology and Stem Cell Laboratories, Monash University, Clayton, Victoria, Australia; 4 Murdoch Childrens Research Institute, Royal Children’s Hospital, Parkville, Victoria, Australia; University of Newcastle upon Tyne, United Kingdom

## Abstract

Mesenchymal progenitors or stromal cells have shown promise as a therapeutic strategy for a range of diseases including heart failure. In this context, we explored the growth and differentiation potential of mesenchymal progenitors (MPs) derived in vitro from human embryonic stem cells (hESCs). Similar to MPs isolated from bone marrow, hESC derived MPs (hESC-MPs) efficiently differentiated into archetypical mesenchymal derivatives such as chondrocytes and adipocytes. Upon treatment with 5-Azacytidine or TGF-β1, hESC-MPs modified their morphology and up-regulated expression of key cardiac transcription factors such as *NKX2-5*, *MEF2C*, *HAND2* and *MYOCD*. Nevertheless, NKX2-5^+^ hESC-MP derivatives did not form contractile cardiomyocytes, raising questions concerning the suitability of these cells as a platform for cardiomyocyte replacement therapy. Gene profiling experiments revealed that, although hESC-MP derived cells expressed a suite of cardiac related genes, they lacked the complete repertoire of genes associated with bona fide cardiomyocytes. Our results suggest that whilst agents such as TGF-β1 and 5-Azacytidine can induce expression of cardiac related genes, but treated cells retain a mesenchymal like phenotype.

## Introduction

Mesenchymal progenitor cells (MPs) have been advanced as a platform for cell based therapy for cardiac repair [1]–[11]. Recently, Mohanty et al reported that bone marrow MPs (BM-MPs) could be induced to express cardiac associated genes by agents such as 5-Azacytidine (5-AZA) and transforming growth factor beta 1 (TGF-β1) [8]. Similarly, MPs could be coerced to undergo cardiomyocyte differentiation by co-culture with neonatal cardiomyocytes [9]. However, it is unclear from these latter studies whether MPs truly adopted a cardiac identity or whether they simply up-regulated a subset of genes associated with cardiomyocytes. Similarly, the ability of MPs to contribute to a functional myocardium in vivo remains an open question.

As an alternative to using functional cardiomyocytes, a number of studies have explored the use of MPs as an adjunct to traditional forms of treatment for heart failure. In this setting, MPs are thought to potentiate the repair process without actively participating in the formation of new cardiomyocytes. However, the limited availability of appropriate MP populations has promoted investigation of hESCs a potential source of MPs. Xu et al. first reported the derivation of fibroblast-like cells from hESCs [12] which they designated human embryonic fibroblast-like cells (HEF1). Since this report, several methods have been investigated for derivation of mesenchymal progenitors from hESCs, including transfection of human telomerase [12], co-culture with OP9 cells [13] or fluorescence-activated cell sorting [14]. Other studies have employed manual selection of mesenchymal-like cell populations [15], [16] or consecutive enzymatic passaging and culture on gelatin or fibronectin in human fibroblast growth factor supplemented media [17], [18]. In each case, the goal of such studies was to generate a uniform population of mesenchymal cells that could be utilized in place of similar cells obtained from *in vivo* sources.

In this study we investigated the potential of hESC derived MPs (hESC-MPs) to undergo cardiac differentiation in response to previously reported cardiogenic stimuli. We developed a reliable and straightforward selective culture method for hESCs derivation into MPs, identified as such by their mesodermal differentiation capacity and marker expression. Treatment with 5-Azacytidine or TGF-β1 induced up-regulation of the expression of some cardiac associated genes. However, no contractile cardiomyocytes were observed suggesting that hESC-MPs have a restricted differentiation capacity akin to that of MPs isolated from other sources [8], [9], [11], [19]–[21]. We provide detailed gene expression profiling and bioinformatic analysis of hESCs, hESC-MPs and hESCs-MPs treated cells. These analysis, provide an explanation as to why these cells did not form functional cardiomyocytes. In conclusion, our results demonstrate that hESC-MPs are a readily expandable MSC-like population but their utility as source of fully functional cardiomyocytes for regenerative medicine requires further investigation.

## Materials and Methods

### hES Cell Lines Used and Culture

Three different cell lines were used for derivation of mesenchymal progenitors. Two cell lines (ES3 and ES4) were purchased from Wicell research institute, and WMC2 -mOrange established in Weill Cornell Medical College (courtesy of Rafii. S.) [22]. The permissions for use of these cell lines were obtained after review by the Cornell-Rockefeller-Sloan Kettering Institute ESC research oversight committee. The funding for execution of these studies was secured from approved non-US federal funding resources. Human ESCs were grown on feeder layer free conditions on growth factor reduced matrigel (#354230, BD biosciences), and cultured with mTeSR1 (#05850, Stemcell Technologies) changed every day. Cultures were performed at 37°C, 5% CO_2_. 1 mg/ml dispase (#07913, Stemcell Technologies) was used for passaging.

### Derivation of hES-MP Cell Lines and Subsequent Expansion

**Figure 1:**
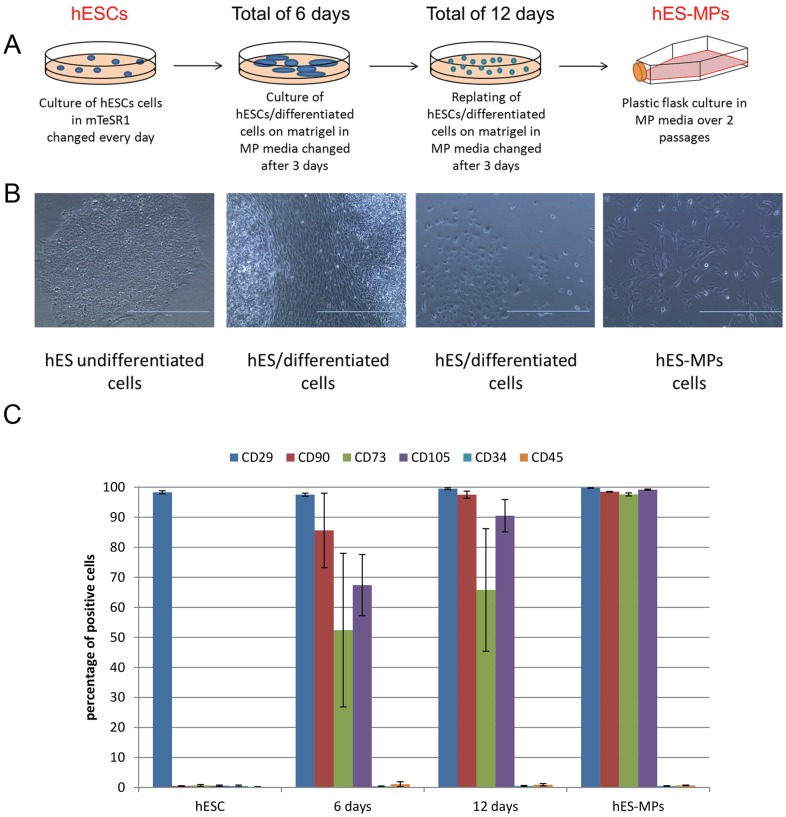
Derivation of hESC-MPs. A. Flow chart of experimental procedure for hESC-MPs derivation. After culture on matrigel in mTESR1 media undifferentiated hESCs were shifted to MP specific media for 6 days. After 6 days the confluent culture of differentiated cells were split to 1/3 with dispase and replated on matrigel. After an extra 6 days of culture, differentiated cells were plated on coating-free feeder-free culture dishes. Cells were analyzed for MP markers expression at each step of the process. B. Phase contrast imaging of cell morphology modification during the derivation process of hESC-MPs. C. Chart representing flow cytometry analysis of mesenchymal progenitor markers CD29, CD90, CD73 and CD105 at the different time-points of our differentiation process.

Undifferentiated hESCs were grown to reach 70% confluence. mTeSR1 was then replaced with MP media (DMEM low glucose with 20% fetal bovine serum (FBS), and 1% penicillin/streptomycin). Differentiation was allowed to proceed for 6 days in this media. Cells were then passaged using dispase (1 mg/ml) and plated on matrigel coated plates for a further six days of culture. Media was changed every 2–3 days. After six days cells were passaged using trypsin and spread on tissue culture treated plastic flasks. All cultures were performed at 37°C, 5% CO_2_. Dead and non-adherent cells were removed the day after initial plating by washing with PBS. Media was then changed every three days. Cells were passaged when reaching 80% confluence. After two passages on plastic, purity of MPs was analyzed by cell surface marker expression, differentiation ability and gene expression. Flow chart of differentiation is given in figure 1A.

### Derivation of hESC-MPs into Cardiomyocyte-like Cells

The established hESC-MPs or BM-MPs (p = 3) were plated at 1.5×10^4^/cm^2^ in 6 well plates. The cells were exposed for 24 h to 10 µM of 5-AZA (Sigma Chemical Co., St. Louis, Missouri, USA) or 10 ng ml^−1^ TGF-β1, (Peprotech Asia, Israel) in MP media. After 24 h the media was changed and cells were propagated in regular BM media for the next 30 days [23], [24]. MPs maintained only in MP media were used as assay control.

### Human Bone Marrow Mesenchymal Stem Cells

Bone marrows MSCs (BM-MPs) were purchased from Stem Cell Inc. (#MSC-001F, Stem Cell Inc.) and maintained in the same culture conditions as hESC-MPs as described above.

### Immunostaining and Fluorescence Activated Cell Sorting (FACS) Analysis

For flow cytometry analysis of cell surface antigens, cells were stained for the expression of CD45, CD34, CD73, CD105, CD90 and CD29 using Mouse anti-human CD45 antibodies coupled with flurochromes as detailed: (BD Biosciences, #339192, clone 2D1) coupled with Amcyan, CD34 (BD Biosciences, #555821, clone 581) coupled with FITC, CD105 (biolegend, #323212, clone 43A3) coupled with AF647, CD73 (BD Biosciences, #550257, clone AD2) coupled with PE, CD29 (biolegend, #323212, clone TS2/16) coupled with APC-Cy7, CD90 (BD Biosciences, #550402, clone 5E10) coupled with AF700. Briefly, 1.10^6^ cells were harvested and non-specific sites were blocked in PBS- 5%FBS-1%BSA-10%FcR Blocking Reagent (Myltenyi Biotec) for 30 minutes on ice. The cell suspension was incubated with specific antibodies for 45 minutes on ice. Intracellular staining was performed using BD Biosciences kit cytofix/cytoperm (#554714) following manufacturer instructions. After washing in PBS and filtration using a 45µm strainer, cells were analyzed by Fluorescence Activated Cell Sorting (FACS) on a SORP FACSAria2 (BD Biosciences) as described below. Data were processed with FACSDiva 6.3 software (BD Biosciences). Doublets were excluded by FSC-W×FSC-H and SSC-W×SSC-H analysis, single stained channels were used for compensation, and fluorophore minus one (FMO) controls were used for gating. [25]. An exemple of dot plot selection is given in figure S1. A minimum of 100,000 events were acquired per sample.

Intracellular staining of cardiomyocyte differentiation proteins was analyzed using a, BD cytofix/cytoperm kit (BD biosciences, #554722) according to manufacturer recommendations. The following primary antibodies were used: Anti-heavy chain cardiac Myosin antibody (abcam, # ab15), Anti-Nkx2.5 (abcam, # ab91196), Anti-alpha smooth muscle Actin (abcam, # ab7817), Anti-NCX1 (Abcam, # ab2869), anti-MRCL3 (Novus, # NBP1-44423), Anti-CTNI (R&D system, #MAB6887), anti-CTNT (abcam, # ab8295). Secondary antibody from Invitrogen (goat anti-mouse IgG1-AF546, # A-21123; IgG2a-AF546, # A-21133; IgG2b-AF488, # A-21141) were used as appropriate. Controls were performed by omitting the primary antibody and applying only the secondary antibody.

### Mesenchymal Lineage Differentiation

All differentiations were performed using human mesenchymal stem cell functional identification kit (#SC006, R&D system), following manufacturer instructions. Briefly, for Osteogenic differentiations: MPs were grown for 2 days in MEM basal media (alpha MEM +10%FSC+Antibiotic), then the media was replaced by differentiation medium (MEM basal media+osteogenic supplement) changed every 3 days. Osteogenic differentiation was assessed by staining cells in wells with Alizarin Red S after 14–21 days.

Adipogenic differentiations: MPs were grown for two days in MEM basal media (alpha MEM +10%FSC+Antibiotic) until reaching 100% confluence, then the media was replaced by differentiation medium (MEM basal media+adipogenic supplement) changed every three days. Adipogenic differentiation was assessed by staining cells in wells with Oil Red O after 10–21 days [26].

### Proteome Array Analysis

Stem cells protein expression was verified in hESCs, hESC-MPs and BM-MPs as control. Human pluripotential stem cell array kit from R&D systems was used for analysis (#ARY010, R&D system) according to manufacturer’s instructions. This experiment was performed on hES4 and its derivatives. Briefly, all cells were cultivated as described, collected and lysed in the lysis buffer provided in the R&D System kit. Proteins were quantified based on sample absorbance at 280 nm using a nanodrop device (Thermo- Scientific). 200 µg of protein were loaded on each array. Arrays were developed using horseradish peroxidase (HRP) and chemiluminescent peroxidase substrate (#CPS1120, Sigma). Data were collected using Geliance CCD camera (Perkin Elmer), and analyzed using ImageJ software (NIH). Array pictures were inverted and background subtracted. We then defined the area for signal capture for all spots as 110–120 micron diameter, using the same area for every spot. We defined our signal as the median pixel density value. For comparison, the independent array values were normalized on their internal positive control intensity values.

### Transcriptomic Analysis

RNA was isolated using the Trizol reagent followed by additional purification using RNAeasy extraction kit from Qiagen (QIAGEN, #74106). Two quality control measures were carried out: (1) A spectrophotometric analysis (2) A size fractionation procedure using a microfluidics instrument (Agilent Technologies). 200 ng of total RNA were analyzed on Affymetrix GeneChip Human Genome U133 Plus 2.0 Array. Data were analyzed using Partek Software (V6.09.1110-6; Affimetrix), Venny online software (BioinfoGP; CNB-CSIC) and Ingenuity Pathway analysis (Ingenuity Systems, Redwood City, CA). Class comparisons between BM-MPs, hESC-MPs, hESCs and hESC-MP-cardiomyocyte (three biological replicates of each) were performed to identify gene expression changes with a significant expression differences (P<0.05) and 2-fold increases or decrease expression.

### Ingenuity Pathway Analysis

We used Ingenuity Pathway Analysis software to identify and analyze relevant pathways from the gene lists obtained from microarray analyses. P values for the enrichment of canonical pathways (calculated by Inguenity Systems software) were generated based on the hypergeometric distribution and calculated with the right-tailed Fisher’s exact t-test for 2×2 contingency tables. Network graphs were constructed by overlaying the genes in the gene list onto a molecular network developed from information contained in the Ingenuity Pathways Knowledge database using keywords such as embryonic stem cells, mesenchymal stem cells and cardiac progenitors. All relationships are supported by at least one reference from the literature, from a textbook, or from canonical information stored in the Ingenuity Pathways knowledge database. We also overlaid the expression data from selected experiments onto the network graphs to illustrate specific genes that may be over or under expressed in particular differentiation states.

### Cell Proliferation Assay

Short term proliferation: hESC-MPs proliferation was compared to BM-MPs for 3 days in culture. Cells were plated at 50000 cells per well in a 6 well plate. Cells were then counted for the following three days. The experiment was performed in triplicate.

Long term proliferation: to monitor hESC-MPs-cardiomyocyte like cells proliferation as compared to hESC-MPs, after 24 h 5-AZA or TGF-β1 treatment (or control), cells were seeded at 50000 cells per well in 6 wells plate, the rest of the cells were plated in a T75 for the next analysis. Cells in each well were counted every two days and a proliferation rate was established every 6 days over 30 days.

### Immunofluorescence

Cells were grown in 8 chamber slides as indicated. Cells were fixed and stained using BD cytofix/cytoperm kit (BD biosciences, #554722) according to manufacturer recommendations. Slides were incubated with primary antibodies 1 h to overnight with Anti-heavy chain cardiac Myosin antibody (abcam, # ab15), Anti-Nkx2.5 (abcam, # ab91196), Anti-alpha smooth muscle Actin (abcam, # ab7817), Anti-NCX1 (Abcam, # ab2869), anti-MRCL3 (Novus, # NBP1-44423), Anti-CTNI (R&D system, #MAB6887). Secondary antibody from Invitrogen (goat anti-mouse IgG1-AF546, # A-21123; IgG2a-AF546, # A-21133; IgG2b-AF488,# A-21141) were used. Slides were mounted with the Fluoromount Kit (Invitrogen). Sections were analyzed with a Zeiss confocal microscope Laser Scanning Microscope 710 (Carl Zeiss). Pictures were analyzed with Zen 2008 V5,0,0228 software (Carl Zeiss).

### Statistical Analysis

For statistical analysis and graphical presentation, Excel (Microsoft Corporation) and SigmaPlot (SysStat, Erkrath, Germany) software was used. Numerical results are given as means ± SEM (n = sample size). The statistical significance was assessed with SigmaPlot or Excel software with a Student’s t test. Statistical significance was accepted for *p<0.05; **p<0.01; ***p<0.001.

## Results

### Derivation and Characterization of hESC-MPs

Our differentiation protocol is based on selection of MPs from a heterogeneous population of differentiated hESCs. hESCs cultured on matrigel coated plates in medium containing FBS gave rise to morphologically distinct differentiated cell types (Figure 1A and B). MPs were subsequently enriched on the basis of their ability to grow rapidly on tissue culture treated plates lacking feeder cells or specific adhesion promoting extracellular substrates. Using this protocol (Figure 1A), we obtained, after two passages, morphologically homogeneous hESC-MPs (Figure 1B).

In order to characterize the hESC-MP population, we performed flow cytometric analysis for mesenchymal markers at different stages of differentiation. Following 6 days differentiation in MP media, cultures contained a substantial population of cells that expressed CD105, CD29, CD90 and CD73 but were negative for CD34 and CD45 (Figure 1C and Figure S1A). Nevertheless, at this time point, the MP-like cells did not survive in feeder and matrigel free cell culture conditions, suggesting they had not yet acquired a mature MP phenotype. After a further 6 days of differentiation (12 in total) 61.4±19.3% of the cells were CD90^+^CD105^+^CD73^+^CD29^+^ (Figure S1B) and CD45^-^CD34^-^. At this stage, a significant number of fibroblast-like cells were able to attach to plastic and survive in MP media in the absence of feeders or adhesion promoting substrates. After two further passages, we obtained a homogenous population of mesenchymal-like cells (hESC-MPs) that expressed CD105, CD73, CD90 and CD29 (>93%) but not CD34 nor CD45 (<0.5%; Figure 1C). BM-MPs displayed a similar pattern and level of expression for these markers (Figure S1C). The expression of these markers remained constant in hESC-MPs for up to 15 passages (data not shown). Similar cell populations were obtained from three different hESC lines demonstrating the reproducibility of our selection process (Figure S1C).

**Figure 2:**
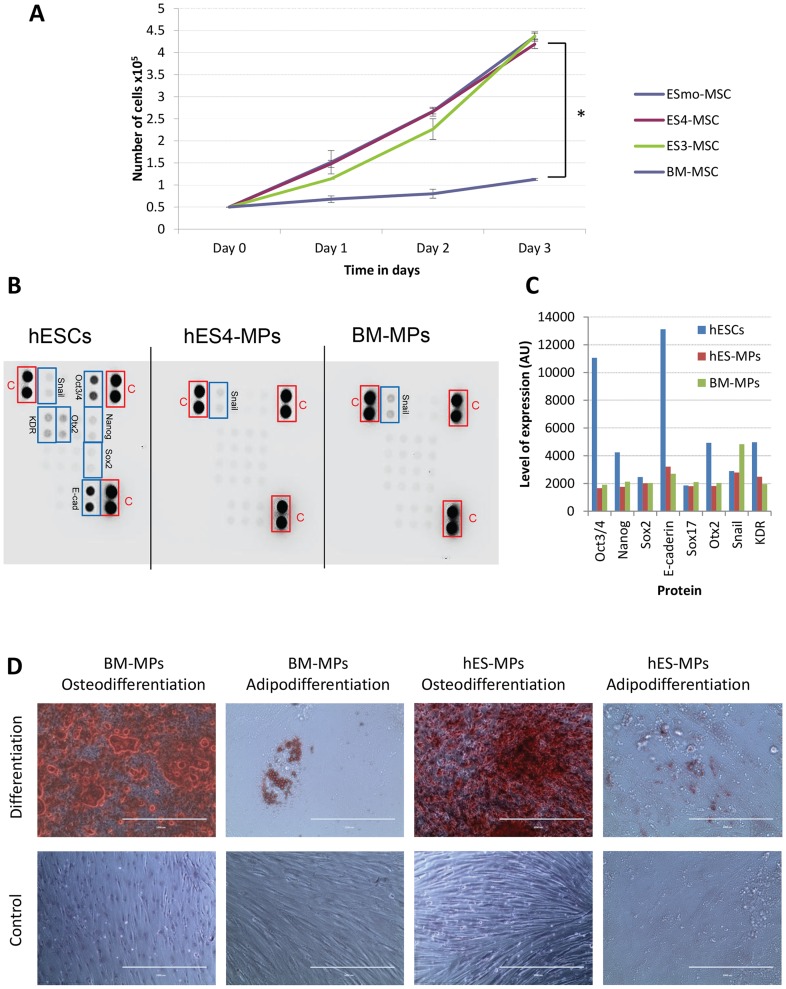
Mesodermal progenitor characterization of hESC derived MPs. A. Growth kinetic comparison over 3 days between BM-MPs and hESC-MPs derived from 3 different hES cell lines (*: p<0.05). B. Pluripotential stem cell array was used to compare protein extracts from hESCs, hES4-MPs and BM-MPs. C. Charts representing the results of the stem cell array. D Phase contrast imaging of the differentiation of hESC-MPs in different mesodermal lineages. Osteoblast were stained using Alizarin Red S and adipocyte with Oil Red O.

The proliferation rate of three independently derived hESC-MP cell lines was significantly higher than BM-MPs (Figure 2A). Moreover, hESC-MPs were expandable up to passage 20 without undergoing changes in morphology or proliferation rate. Commercially obtained BM-MPs were quiescent by passage 8 to 10 in our culture conditions.

In contrast to hESCs, hESC-MPs lacked expression of *OCT4*, *NANOG*, *ECAD* and *OTX2* and displayed a similar profile to BM-MPs (Figure 2 B,C). Like BM-MPs, hESC-MPs were able to differentiate into mesenchymal derivatives such as adipocytes and osteoblasts (Figure 2D). Thus, cell surface marker analysis and mesodermal lineage differentiation confirmed the mesenchymal phenotype of hESC-MP cells.

### Potential of hESC-MPs to Differentiate to Cardiomyocytes

**Figure 3:**
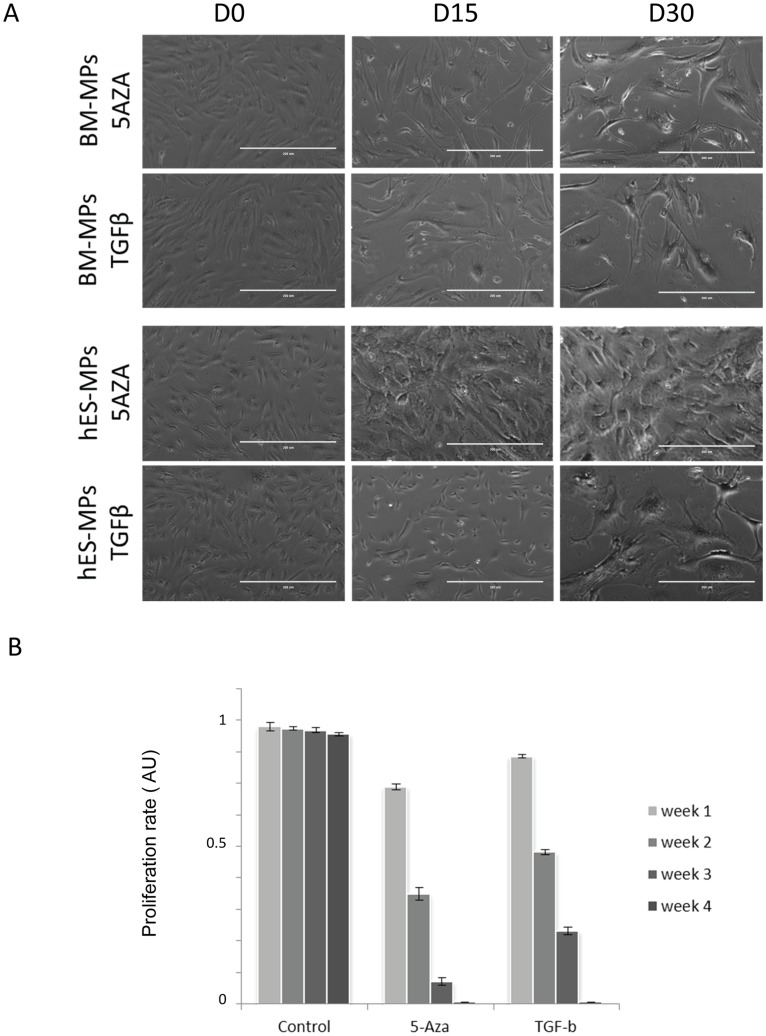
MPs modify their morphology and proliferation upon treatment with 5AZA or TGF-β1. A. Phase contrast imaging of the morphological modification of the hES-MPs or the BM-MPs after treatment with 5-AZA (10 µM )or TGF-β1 (10 ng ml−1). B. Proliferation of hESC-MPs treated with 5-AZA or TGF-β1 was evaluated using cell counting, charts demonstrating the reduction in proliferation over 4 weeks of treatment.

Previous reports identified 5-AZA or TGF-β1 as agents that could induce murine and, more recently, human BM-MPs to up-regulate the expression of genes associated with cardiomyocytes [8]–[10]. We observed that hESC-MPs treated with TGF-β1 or 5-AZA, adopted a more flattened and round morphology compared with controls (Figure 3A). This change in morphology was accompanied by a decreasing proliferation rate, with cells effectively becoming quiescent 4 week after treatment (Figure 3B). Withdrawal from the cell cycle was more rapid in 5-AZA treated cultures for both BM-MPs and hESC-MPs.

**Figure 4:**
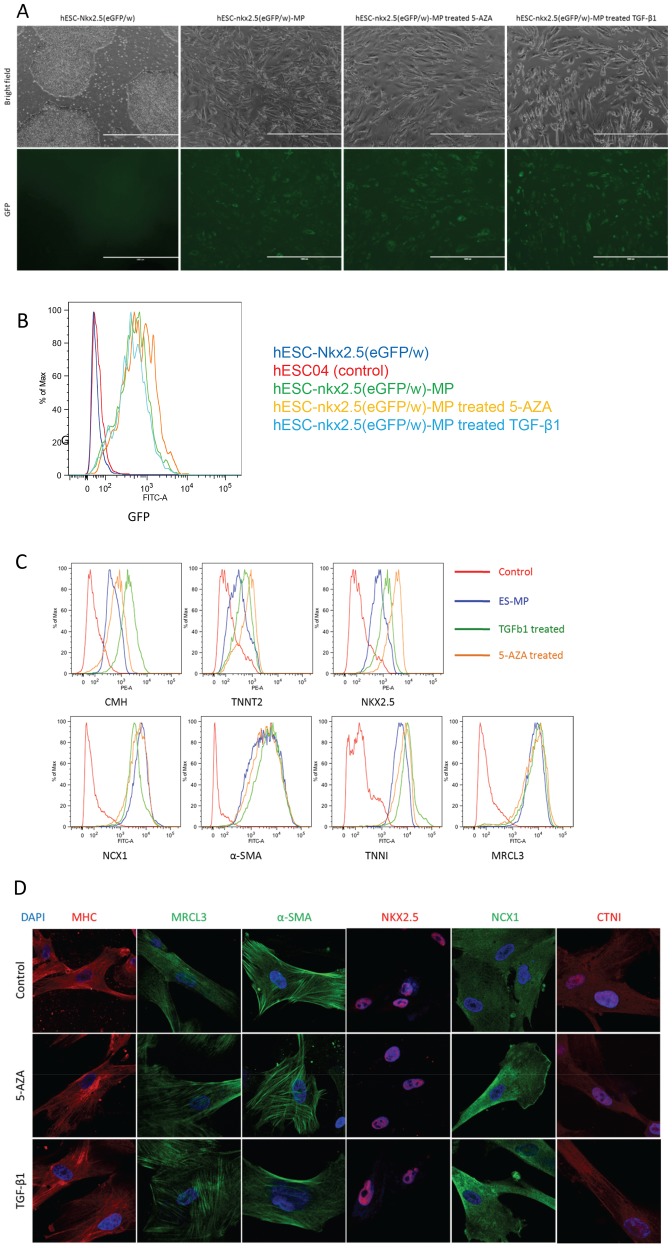
Cardiac marker expression on hESC-MPs and 5-AZA or TGF-β1 treated cells. A. Phase contrast microscopy of hESC-NKX2.5 cell line submitted to our MP derivation protocol demonstrating GFP expression reporting NKX2.5 in our MPs. 5-AZA and TGF-β1 treatments do not modify the GFP expression (last two panels). B. Overlay representing GFP expression (reporting NKX2.5) at day 30 of treatment by 5-AZA or TGF-β1. C. Overlay representing flow cytometry analysis of cardiac myosin heavy chain (CMH), Cardiac Troponin T (CTNT), Homeobox protein NKX2.5, The sodium-calcium exchanger (NCX1), alpha smooth muscle actin (α-SMA), Cardiac Troponin I (CTNI) and Myosin regulatory light chain (MRCL3) in hES-MPs untreated or treated for 30 days by 5-AZA or TGF-β1. D. Confocal microscopy imaging of the expression of cardiac marker genes. MHC, MRCL3, αSMA demonstrate a striated pattern. CTNI demonstrated a weak striated pattern. NKX2.5 is clearly labeled in the nucleus. (x 600).

To better evaluate the cardiac potential of our hESC-MPs, we employed *NKX2-5^eGFP/w^* hESCs in which GFP reports the expression of the cardiac associated transcription factor NKX2-5 [27]. MPs derived from *NKX2-5^eGFP/w^* hESCs displayed weak GFP expression by fluorescence microscopy; a result confirmed by flow cytometry (Figure 4A and 4B). 5-AZA and TGF-β1 treatments induced growth arrest and morphological changes as with hESC-MPs, however, consistent with our analyses described above, no significant increase in GFP expression was observed (Figure 4A and 4B and Figure S2).

These results were mirrored when genetically un-modified hESC lines were used as a source of hESC-MPs. Intracellular flow cytometry confirmed that the altered morphology and decreased proliferation we observed in response to TGF-β1 or 5-AZA treatment correlated with the onset of expression of cardiac markers. This analysis demonstrated expression of NKX2-5, cardiac myosin heavy chain (CMH), cardiac myosin light chain (MRCL3), CTNI, CTNT, NCX1, and α-smooth muscle actin (α-SMA) in hESC-MPs. Whilst TGF-β1 and 5-AZA treated cells also expressed these markers (Figure 4C), no statistically significant difference was found between the expression levels of these genes in hESC-MPs and 5-AZA or TGF-β1 treated cells. Flow cytometric data was confirmed for a subset of proteins by immunofluorescence. This analysis showed that the myofilament proteins, MHC, MRCL3 and α-SMA displayed a striated pattern (Figure 4D) often associated with cardiomyocytes. However, CTNI was more diffuse and not restricted to the myofilament network. As expected, NKX2-5 was restricted to the nuclei in all three cells types (Figure 4D, middle panels).

Overall, while hESC-MPs treated with 5-AZA or TGF-β1 expressed some cardiac associated genes, they do not form fully functional cardiomyocytes: a conclusion consistent with previous publications [8], [10], [12]–[14].

### Transcriptomic Analysis of hESCs, hESC-MPs and BM-MPs

**Figure 5:**
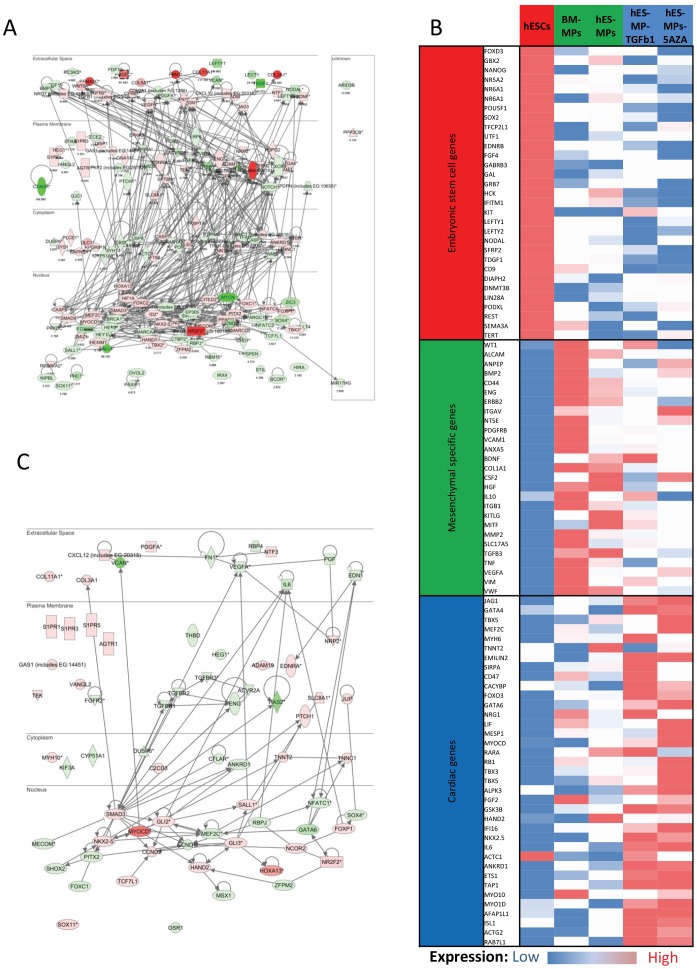
Level of expression of embryonic stem cell, mesenchymal and cardiac specific genes in hESC-MPs. A. Genes involved in cardiomyocyte development were retrieved using IPA canonical gene list from the transcriptomic analysis of hESC-MPs compared to hESCs. Network representing expression and cellular localization of the genes retrieved. Up-regulated (red), down-regulated (green) in hESC-MPs compared to hESCs. B. Schematic representation of level of expression of selected genes (red: higher expression level; blue: lower level of expression). While embryonic stem cells genes are overexpressed by hESCs; hESC-MPs and BM-MPs overexpress mesenchymal genes and cardiac markers are more abundant in 5-AZA or TGF-β1 treated cells. C. Network representing expression and cellular localization of genes involved in cardiomyocyte differentiation, up-regulated (red) or down-regulated (green) in hESC-MPs compared to BM-MPs.

**Figure 6:**
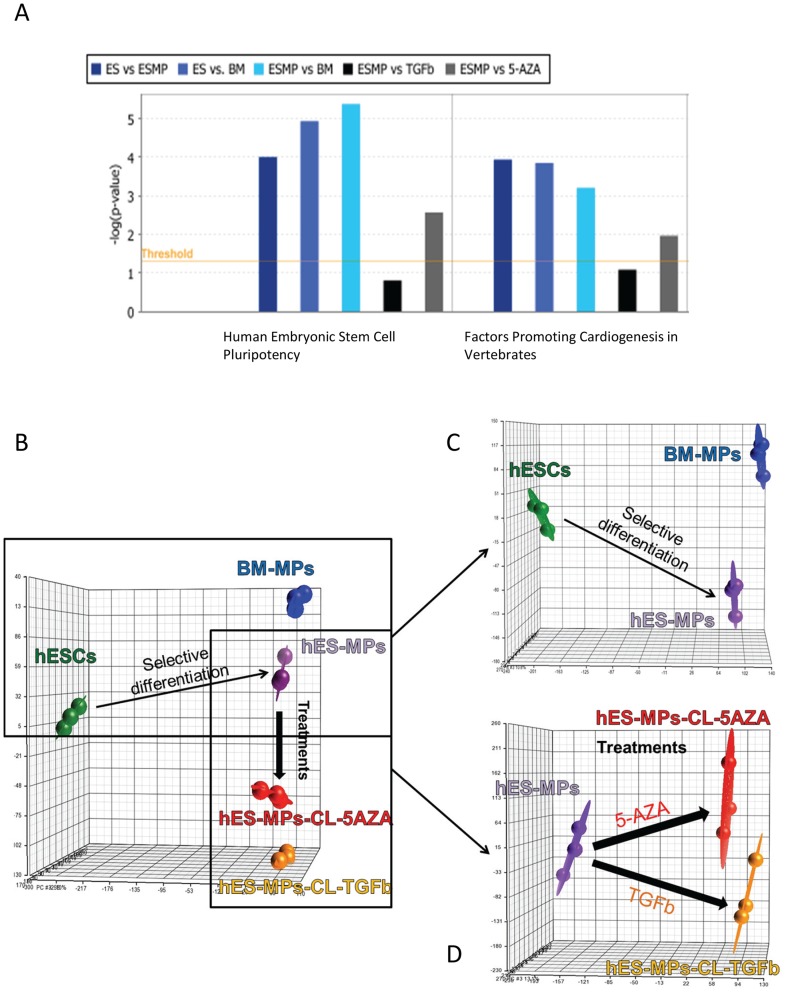
Transcriptomic comparison of hESCs, BM-MPs and hESC-MPs. A. Pathway enrichment analysis (Ingenuity Pathway) of gene expression data shows the significance of gene expression changes between different differentiation states in two selected pathways. The threshold is set at a p-value of 0.05. B-D. Schematics of PCA analysis demonstrating the relationship between the different cell types.

Given the absence of functional cardiomyocyte differentiation observed in our 5-AZA or TGF-β1 treated cultures, we were interested to determine if this outcome reflected an underlying deficit in the repertoire of genes expressed by the end stage cell populations. In the first instance, we analyzed the expression of cardiogenic transcription factors in hESC-MPs compared to hESCs. Consistent with our prior flow cytometry studies, this analysis confirmed that hESC-MPs expressed the key cardiac transcription factor NKX2-5. Moreover, our gene expression data demonstrated that multiple genes with known roles in cardiac differentiation were up-regulated in hESC-MPs, including *TNNT2*, *MEF2C*, *MYOCD*, *HAND2*, *ANKRD1*, *IFI16*, *TBX3*, *CCND1*, *PITX2*, *LIF*, *GSK3B*, *NRG1*, *ITBG1*, *ACTN1*, *ADM*, *FOXP1* and *RXRB* (Figure 5A and Figure 5B). In addition, pathway enrichment analysis of gene expression showed that cardiac-related genes were more highly expressed in hESC-MPs compared to BM-MPs, possibly suggesting the former may have an increased propensity for cardiomyocyte differentiation. Critically, many of these genes encoded transcription factors required to drive cardiomyocyte differentiation (e.g *NKX2-5, MEF2C, MYOCD*; Figure 5C and Figure 6A) [28].

Principal component analysis (PCA) demonstrated that hESC-MPs had a significantly different transcriptomic signature from hESCs (Figure 6B, C). Pathway enrichment analysis (Figure 6A) also showed a significant difference in the expression of genes affecting pluripotency between hESCs, hESC-MPs and BM-MPs. Indeed, 9001 genes showed at least a 2-fold change in expression levels between hESCs and hESC-MPs (p = 0.05, FDR = 0.01). Similarly, 10561 genes were differentially expressed between hESCs and BM-MPs. Consistent with our pluripotency marker analysis, hESC-MPs expressed dramatically lower levels of key pluripotency genes, including the transcription factors *SOX2*, *OCT4*, *NANOG* (all >100 fold down-regulated) and other embryonic stem cell markers (Figure 6A and Figure S3 and 4A). In contrast, hESC-MPs showed elevated expression of a suite of mesenchymal-specific genes (Figure 5B and Figures S3 and 4B), including classical MSC markers such as *VCAM1, ALCAM, CD44, ENG, MCAM, NT5E, PDGFRB*, and other genes associated with MSCs such as *ANXA5, COL1A1, ICAM1, IL10, IL6, ITGB1, TGFB3, VEGFA and VIM* (Figure S3). Finally, expression of genes implicated in epithelial to mesenchymal transition (EMT) was also increased in hESC-MPs relative to hESCs. For example, elevated levels of *N-CAD* (fold >7) and *TWIST1* (fold >44) in hESC-MPs correlated with loss of *E-CAD* expression during differentiation from hESCs (see Figure S4C). Therefore, the gene expression profile of hESC-MPs is consistent with a proposition that these cells have undergone a shift from a totipotent state toward a lineage restricted mesenchymal state.

BM-MPs had a distinct gene expression signature compared with both hESCs and hESC-MPs (Figure 5B, 6B and 6C). However, BM-MPs were more closely related to hESC-MPs, sharing 5232 differentially expressed transcripts (compare to hESCs) (Figure S5A). Genes enrichment analysis revealed that BM-MPs were enriched for transcripts associated with differentiation and specification of mesenchymal cells (Figure 5B). Furthermore, although there were 2031 mRNAs displaying a greater than two fold expression differential between hESC-MPs and BM-MPs, PCA and unsupervised hierarchical clustering analysis confirmed the close resemblance of these two cell types (Figure 5B, 6B and 6C). Ingenuity analysis of the genes differentially expressed between BM-MPs and hESC-MPs demonstrated an enrichment for genes involved in bone, connective tissue and fibroblast development in the former (Figure S4D, E and F). A list of the most significantly differentially expressed genes (fold change >20) is given in table S1.

Transcriptomic analysis was performed on differentiated hESC-MPs at 30 days post treatment with 5-AZA or TGF-β1. PCA analysis clearly demonstrated that hESC-MPs treated with 5-AZA (hESC-MP-5AZA-CL; CL denotes cardiomyocyte-like) and TGF-β1 (hESC-MP-TGFb1-CL) represented transcriptionally distinct cell types when compared to the parental hESC-MPs (Figure 6B and 6D). Indeed, 1507 and 1598 genes were differentially expressed between hESC-MPs-TGFb1-CL and hESC-MPs-5AZA-CL when compared to hESC-MPs, respectively. Further, 1247 genes were commonly differentially expressed in both differentiated cells (Figure S5B). A list of these differentially expressed genes is given in table S2. Interestingly, when compared with one another, hESC-MP-5AZA-CL and hESC-MP-TGF-β1-CL displayed only 17 differentially expressed genes. Known cardiomyogenesis/myogenesis markers *MYO1D*, *ACTG2*, *ACTC1*, *MYLIP*, *MYO10*, *MyoZ2*, *MYPN* and *JAG1* were acquired during differentiation induced with both treatments. However, there was no alteration in the levels of expression of major cardiac transcription factors. This analysis demonstrated that both treatments induced similar transcriptomic reprogramming in hESC-MPs.

Pathway analysis of those genes differentially expressed between hESC-MPs and their differentiated derivatives indicated down-regulation of genes implicated in cell cycle, consistent with the loss of cell proliferation observed in our cultures following treatment (Figure S6A and S6B). Intriguingly, despite the absence of contractile cells, this analysis also revealed up-regulation of a series of genes implicated in contractility (*ACTC1*, *ACTG2*) (Figure S6C and S6D) together with some genes implicated in promoting cardiogenesis (*NOG*, *FZD8*, *TGFB2*) (Figure S6E and S6F).

The lack of contractile cells prompted us to investigate if the cells generated following 5-Aza or TGF-β1 treatment were cardiac fibroblasts, a cell type previously identified from *in vivo* sources that is known to express cardiac associated genes. Indeed, cardiac fibroblasts are the second most abundant cell in the heart and share common gene expression with cardiomyocytes (*VIM*, *GATA6*, *POSTN*, *MEF2C*, *COLLA1*, *COLLA2*, *DDR2*, *FLNA*, *TNC*) [29], [30]. Those genes were found over-expressed in our hESC-MPs and 5-Aza or TGF-β1 treated cells when compared to hESC. FSP1 (also named S100A4) is also overexpressed in BM-MPs and hESC-MPs when compared to hESCs. However, following 5-Aza and TGF-β1 treatments, POSTN, COLLa2, TNC and FSP1 were significantly down-regulated. This indicates that whilst hESC-MPs together with BM-MPs do share some common markers with cardiac fibroblasts, exposure to 5-Aza and TGF-β1 does not induce specific differentiation toward cardiac fibroblasts.

## Discussion

We developed a simple method for generating mesenchymal progenitor cells from hESCs. After 12 days of culture in MP medium, we generated fibroblastic like cells that gave rise to a homogeneous mesenchymal progenitor population. This protocol capitalized on the enhanced proliferation rate of mesenchymal progenitor cells relative to other cells present within these cultures. Cell surface marker analysis together with mesenchymal lineage differentiation, confirmed the identity of these cells as mesenchymal progenitors (hESC-MPs). Our procedure took advantage of human embryonic stem cells to differentiate into fibroblast like cells in presence of serum. For example, Stojkovic and colleagues [31] demonstrated that fibroblast like cells, designated human ESC-derived fibroblasts, formed spontaneously in hESC cultures. Although they showed these cells expressed MSC surface markers such as CD44 and CD90, their differentiation potential into mesenchymal lineages was not further investigated. We hypothesized that it was possible to accelerate the differentiation process by switching our hESC culture from serum free to serum containing medium. Indeed, hESCs underwent a morphological change as early as the first day following transfer to MP culture conditions.

We assessed the potential hESC-MPs in cardiac differentiation protocols. Two protocols were used based on studies in the mouse system: a method employing 5-Azacytidine [8]–[10], [24], [32]–[35] and a second protocol using TGF-β1 [8]. 5-AZA has been used to promote cardiomyocyte differentiation of mouse ES cells [36], murine MPs [24], [32]–[35] and human BM-MPs [8]–[10]. In line with published work, both 5-AZA and TGF-β1 treatments of hESC-MPs induced morphological alterations and changes in gene expression. hESC-MPs showed elevated expression of key cardiac markers/transcription factors *NKX2-5*, *MEF2C*, *HAND2* and *MYOCD*, and proteins important for cell contraction like light and heavy cardiac myosin, ACTA2, ACTN1, TNNI or NCX1. Given that hESC-MPs already expressed a large number of cardiac associated genes (*NKX2-5* expression among others), we hypothesized that these progenitors might have served as an ideal starting point for the generation of fully functional cardiomyocytes. This idea was also reinforced by the recent finding that hESC-MPs, unlike adult MPs (BM-MPs), display intrinsic electrophysiological action potential [20]. Nevertheless, contractile cell populations were never observed following differentiation of hESC-MPs with either 5-AZA or TGF-β1, suggesting that neither treatment was able to promote the formation of genuine cardiomyocytes from this progenitor pool.

Cell transplantation is an emerging therapeutic approach for cardiac injury. Cell-based treatments using adult derived stem cells have had encouraging outcomes in animal models [37]–[39]. Promising results have also been obtained in the treatment of cardiac injury using mesenchymal progenitors [40], [41]. In addition, a number of animal studies have shown the potential of BM-MPs to differentiate towards cardiomyogenic lineage [8], [42]–[44]. In this study we established a straightforward procedure for the derivation of mesenchymal progenitors from hESCs. Although these progenitors express many cardiac markers, differentiation of these cells into functional cardiomyocytes remains elusive. However, given that adult derived MPs have already been demonstrated as potentially helpful for cardiac regeneration [45], [46], the hESC-MPs described in this study may prove useful even if further cardiac differentiation *in vitro* is difficult to obtain.

## Supporting Information

Figure S1 Mesenchymal marker expressions at different time-points of the MP derivation process. A. Dot Plot representing CD73, CD29, CD105 and CD90 staining in hESCs and the differentiation derivates at different time-points. B. Dot plot representing CD45, CD34, CD73, CD29, CD105 and CD90 at day 12 of differentiation. C. Charts displaying the different marker intensity for the different cell lines studied after two passages.
(TIF)

Figure S2 Dot plot showing the gating strategy to analyze GFP expression for hESC-NKX2-5eGFP/w-MPs.
(TIF)

Figure S3 Stem cell and mesenchymal specific markers expression were compared between hESCs, BM-MPs or hESC-MPs. A. Embryonic stem cell genes are overexpressed by hESCs when compared to hESC-MPs and BM-MPs. B. Mesenchymal stem cell markers are over expressed in hESC-MP and BM-MPs compare to hESCs. Folds are given between brackets.
(TIF)

Figure S4 Ingenuity pathway analysis of differentially expressed genes between hESCs, hESC-MPs and BM-MPs. A. Network of genes involved in stemness in hESC-MPs when compared to hESCs. As expected the major genes responsible for stemness are downregulated (green) in hESC-MPs when compared to hESCs. B. Network of genes involved in the mesoderm development are up-regulated (red) or down-regulated (green) in hESC-MP when compared to hESCs. C. Network of genes involved in epithelial to mesenchymal transition up-regulated (red) or down-regulated in hESC-MPs when compared to hESCs. D. Network of genes involved in bone development up-regulated (red) or down-regulated (green) in BM-MPs when compared to hESC-MPs. E. Network of genes involved in connective tissue development up-regulated (red) or down-regulated (green) in BM-MPs when compared to hESC-MPs. F. Network of genes involved in development of fibroblast up-regulated (red) or down-regulated (green) in BM-MPs when compared to hESC-MPs.
(TIF)

Figure S5 Venn diagram. A. A large majority of gene differentially expressed between hESC/hESC-MPs and hESC/BM-MPs are shared between hESC-MPs and BM-MPs (5235 out of 7675) B. Similarly a large majority of genes (1247 out of 1858) are commonly differentially expressed between 5-AZA and TGF-β1 treated cells when compared to hESC-MPs.
(TIF)

Figure S6 Ingenuity pathway analysis of differentially expressed genes between hESC-MPs and 5-AZA or TGF-β1 treated cells. A-B. Network of genes involved in “cell cycle”. C-D. Network graphs of genes implicated in contractility. E-F. Network graphs of genes involved the promotion of cardiogenesis. (red) upregulated. (green) downregulated.
(TIF)

Table S1. (XLSX)

Table S2. (XLS)
