# Supplementary figures and images for: Human Embryonic Stem Cell Derived Mesenchymal Progenitors Express Cardiac Markers but Do Not Form Contractile Cardiomyocytes
Source: PLoS One. 2013 Jan 16;8(1):e54524. doi: 10.1371/journal.pone.0054524 (PMC3546995; doi:10.1371/journal.pone.0054524)

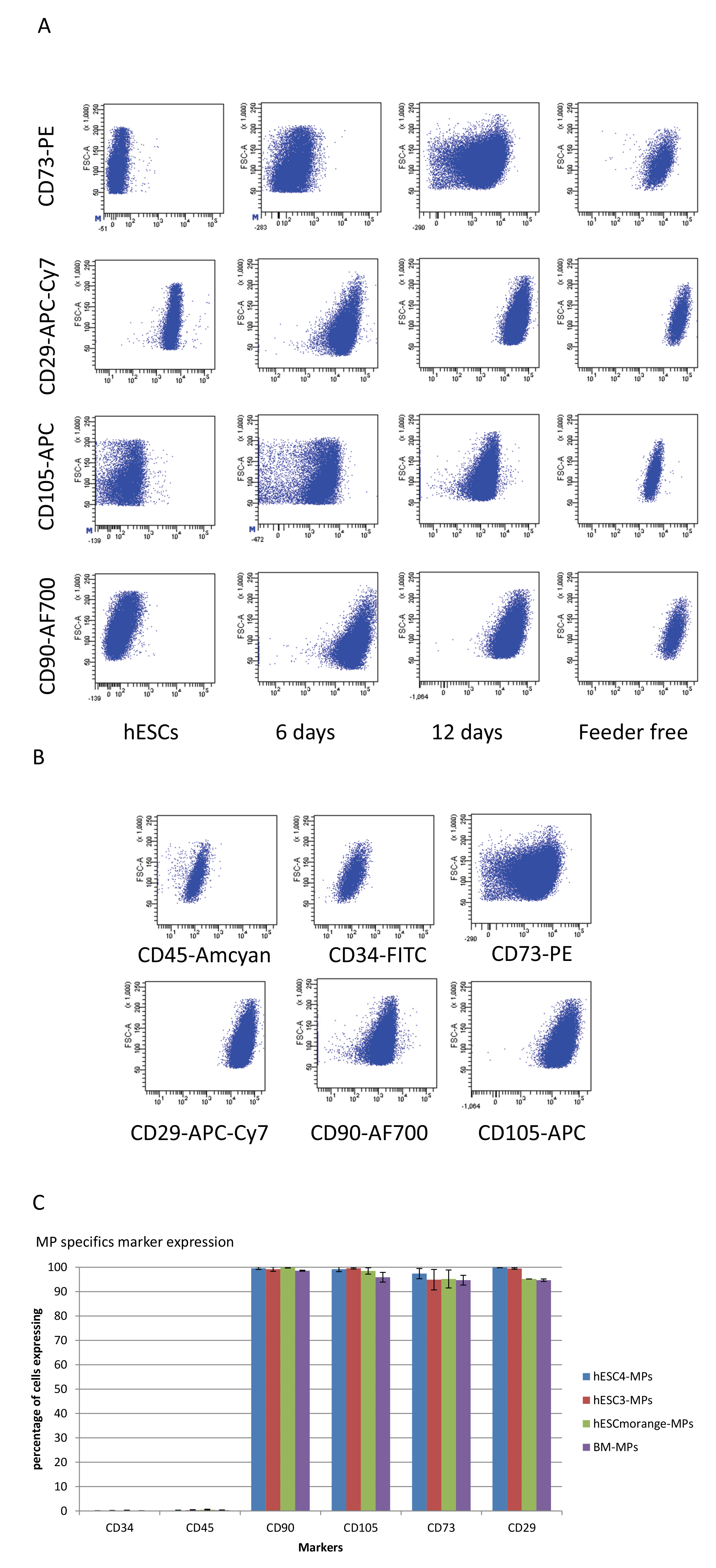

Supplement: Figure S1 — Mesenchymal marker expressions at different time-points of the MP derivation process. A. Dot Plot representing CD73, CD29, CD105 and CD90 staining in hESCs and the differentiation derivates at different time-points. B. Dot plot representing CD45, CD34, CD73, CD29, CD105 and CD90 at day 12 of differentiation. C. Charts displaying the different marker intensity for the different cell lines studied after two passages. (TIF) [file pone.0054524.s001.tif]

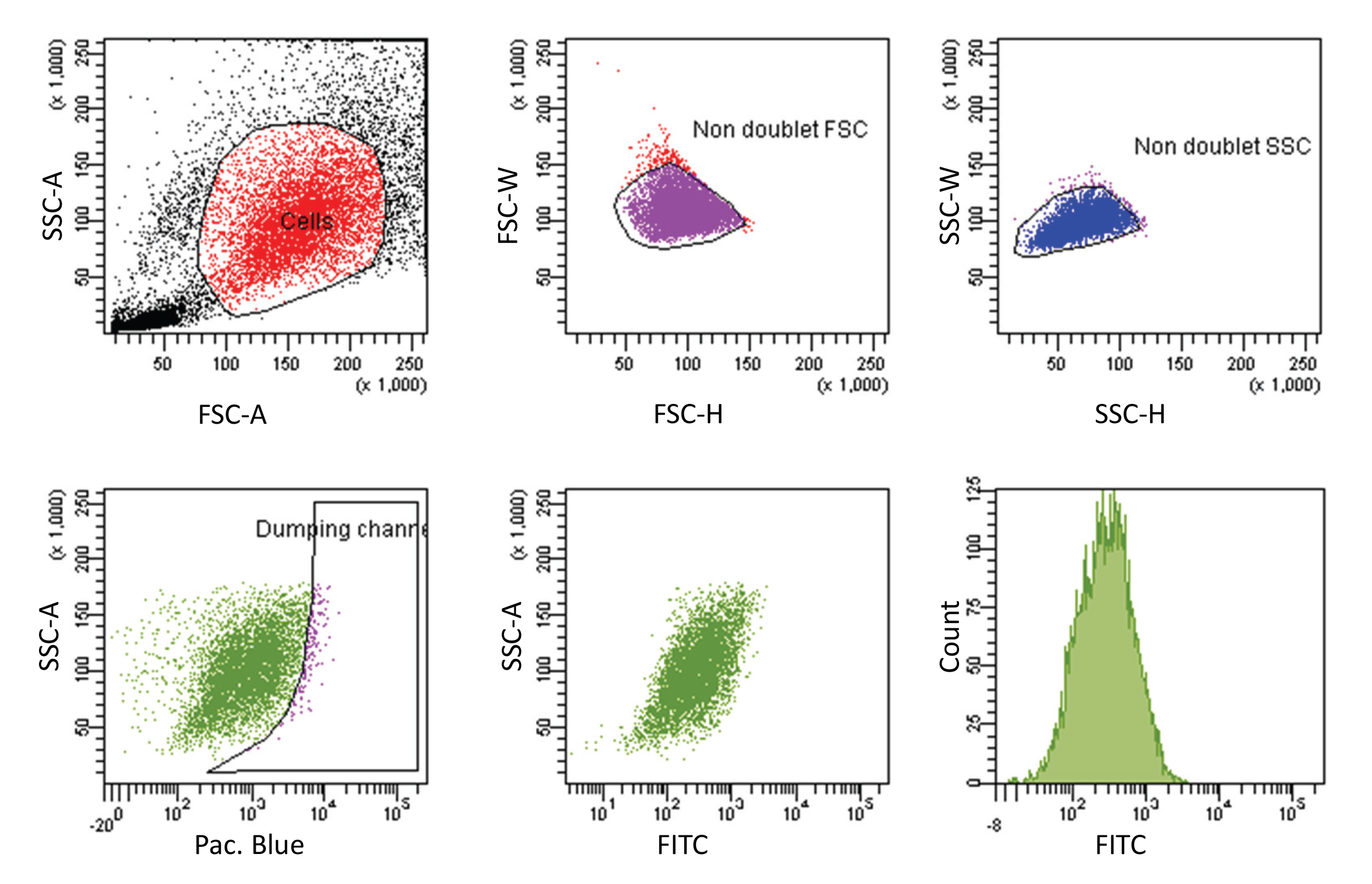

Supplement: Figure S2 — Dot plot showing the gating strategy to analyze GFP expression for hESC-NKX2-5eGFP/w-MPs. (TIF) [file pone.0054524.s002.tif]

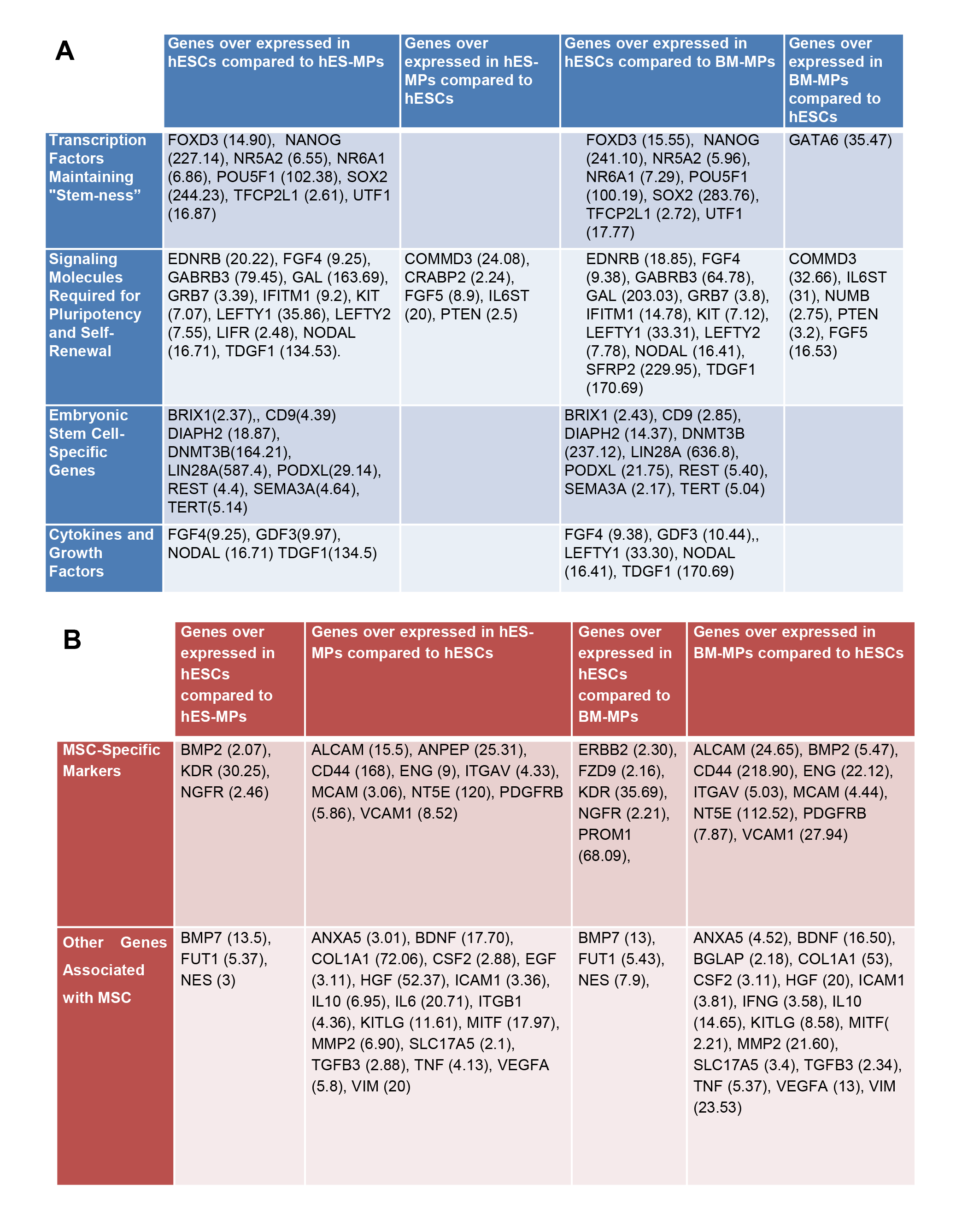

Supplement: Figure S3 — Stem cell and mesenchymal specific markers expression were compared between hESCs, BM-MPs or hESC-MPs. A. Embryonic stem cell genes are overexpressed by hESCs when compared to hESC-MPs and BM-MPs. B. Mesenchymal stem cell markers are over expressed in hESC-MP and BM-MPs compare to hESCs. Folds are given between brackets. (TIF) [file pone.0054524.s003.tif]

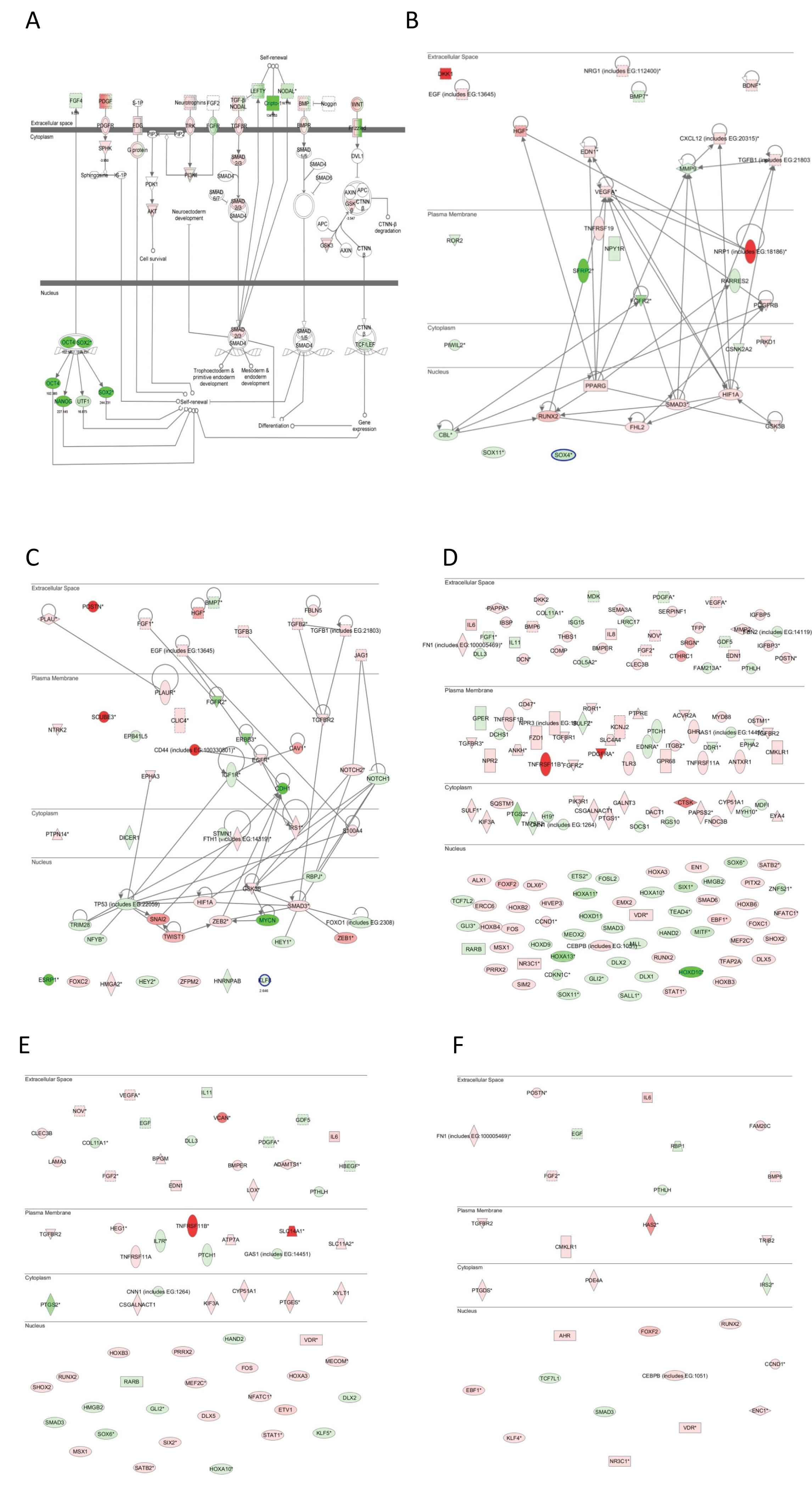

Supplement: Figure S4 — Ingenuity pathway analysis of differentially expressed genes between hESCs, hESC-MPs and BM-MPs. A. Network of genes involved in stemness in hESC-MPs when compared to hESCs. As expected the major genes responsible for stemness are downregulated (green) in hESC-MPs when compared to hESCs. B. Network of genes involved in the mesoderm development are up-regulated (red) or down-regulated (green) in hESC-MP when compared to hESCs. C. Network of genes involved in epithelial to mesenchymal transition up-regulated (red) or down-regulated in hESC-MPs when compared to hESCs. D. Network of genes involved in bone development up-regulated (red) or down-regulated (green) in BM-MPs when compared to hESC-MPs. E. Network of genes involved in connective tissue development up-regulated (red) or down-regulated (green) in BM-MPs when compared to hESC-MPs. F. Network of genes involved in development of fibroblast up-regulated (red) or down-regulated (green) in BM-MPs when compared to hESC-MPs. (TIF) [file pone.0054524.s004.tif]

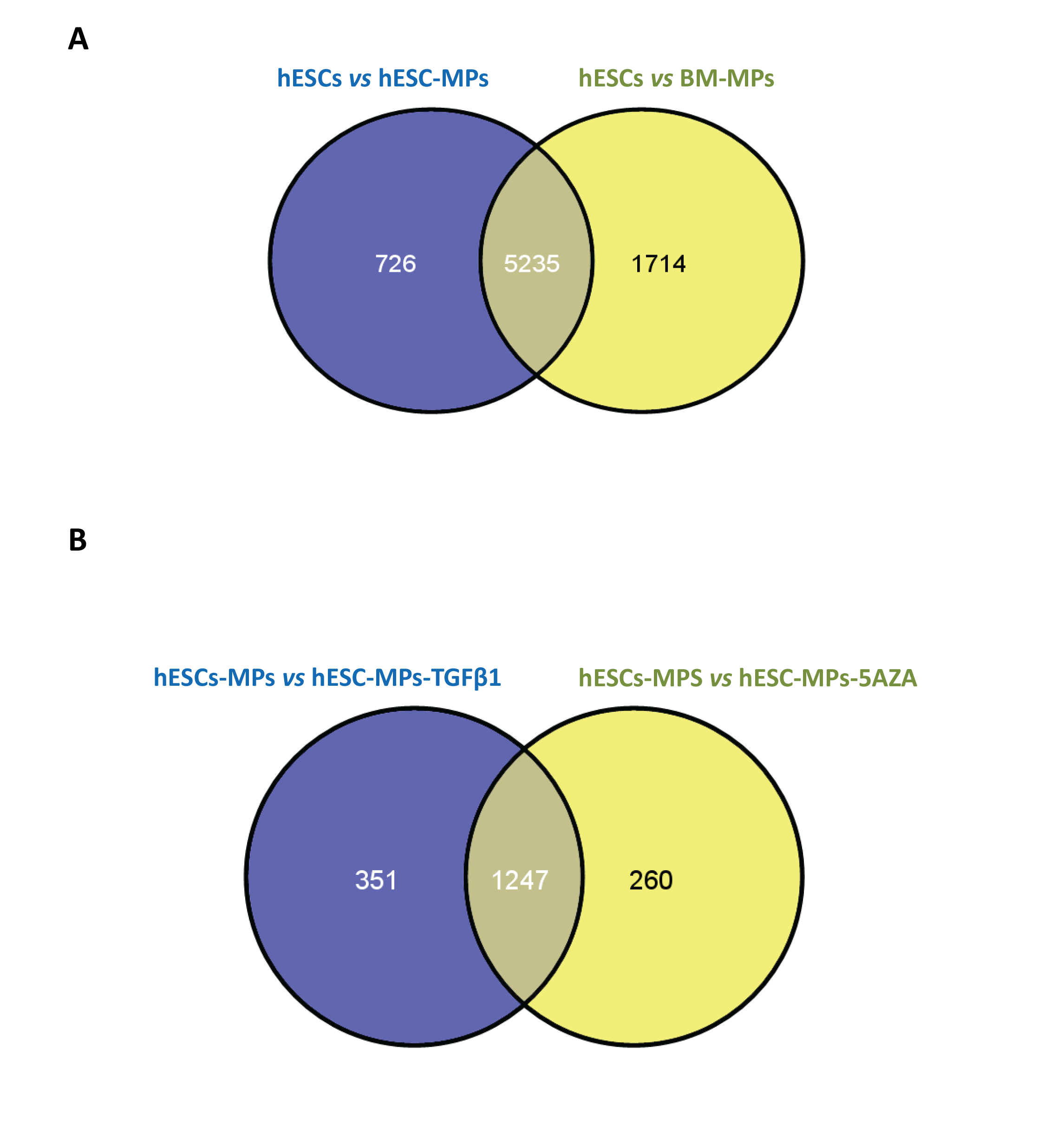

Supplement: Figure S5 — Venn diagram. A. A large majority of gene differentially expressed between hESC/hESC-MPs and hESC/BM-MPs are shared between hESC-MPs and BM-MPs (5235 out of 7675) B. Similarly a large majority of genes (1247 out of 1858) are commonly differentially expressed between 5-AZA and TGF-β1 treated cells when compared to hESC-MPs. (TIF) [file pone.0054524.s005.tif]

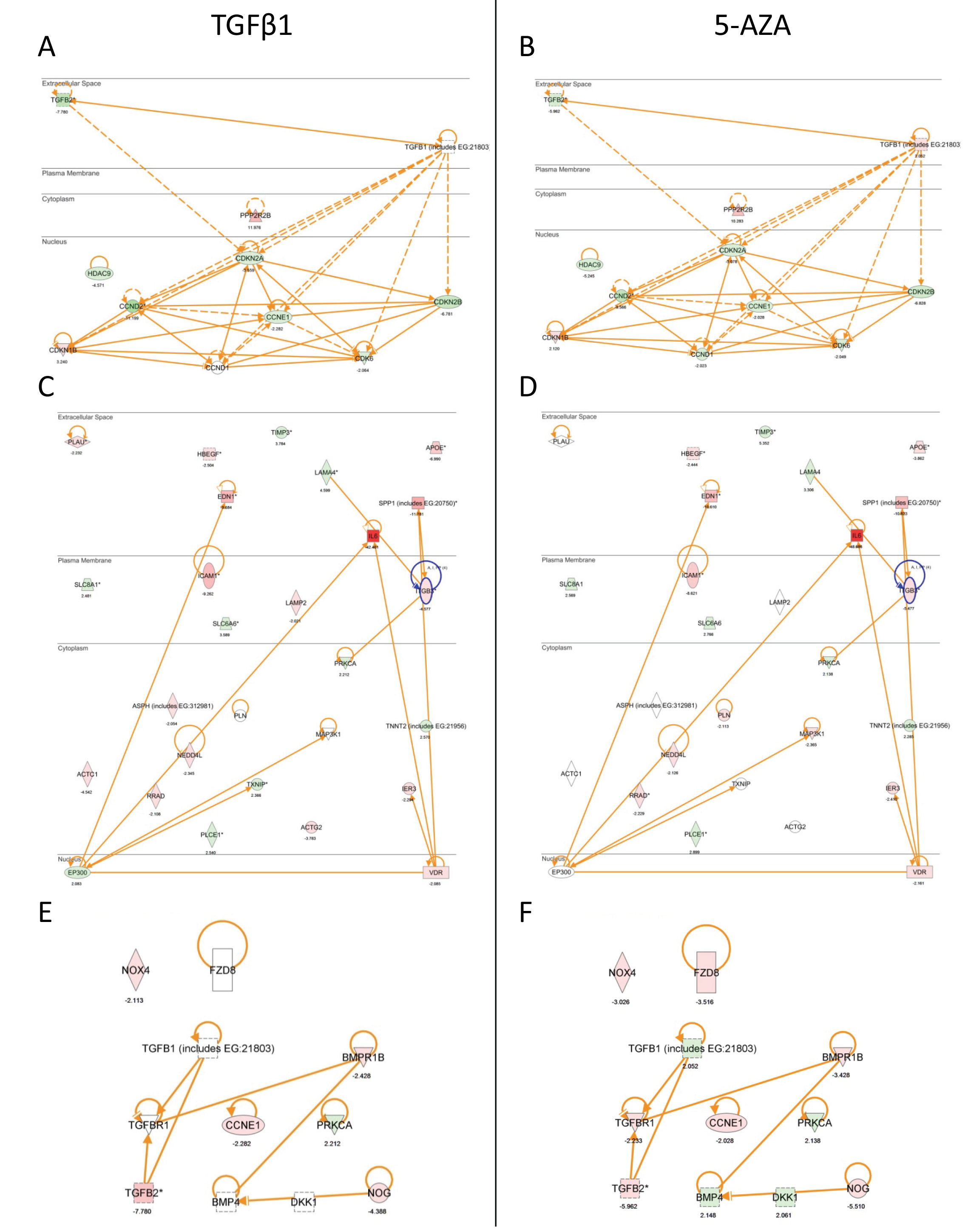

Supplement: Figure S6 — Ingenuity pathway analysis of differentially expressed genes between hESC-MPs and 5-AZA or TGF-β1 treated cells. A-B. Network of genes involved in “cell cycle”. C-D. Network graphs of genes implicated in contractility. E-F. Network graphs of genes involved the promotion of cardiogenesis. (red) upregulated. (green) downregulated. (TIF) [file pone.0054524.s006.tif]
